# Adverse Intrauterine Environment and Cardiac miRNA Expression

**DOI:** 10.3390/ijms18122628

**Published:** 2017-12-06

**Authors:** Mitchell C. Lock, Kimberley J. Botting, Ross L. Tellam, Doug Brooks, Janna L. Morrison

**Affiliations:** 1 Early Origins of Adult Health Research Group; School of Pharmacy & Medical Sciences, Sansom Institute for Health Research, University of South Australia, Adelaide, SA 5001, Australia; mitchell.lock@mymail.unisa.edu.au (M.C.L.); kb555@cam.ac.uk (K.J.B.); ross.tellam@gmail.com (R.L.T.); 2 CSIRO Agriculture, 306 Carmody Rd, St. Lucia, QLD 4067, Australia; 3 Mechanisms in Cell Biology and Disease Research Group School of Pharmacy & Medical Sciences, Sansom Institute for Health Research, University of South Australia, Adelaide, SA 5001, Australia; doug.brooks@unisa.edu.au

**Keywords:** miRNA, epigenetics, heart disease, fetal development

## Abstract

Placental insufficiency, high altitude pregnancies, maternal obesity/diabetes, maternal undernutrition and stress can result in a poor setting for growth of the developing fetus. These adverse intrauterine environments result in physiological changes to the developing heart that impact how the heart will function in postnatal life. The intrauterine environment plays a key role in the complex interplay between genes and the epigenetic mechanisms that regulate their expression. In this review we describe how an adverse intrauterine environment can influence the expression of miRNAs (a sub-set of non-coding RNAs) and how these changes may impact heart development. Potential consequences of altered miRNA expression in the fetal heart include; Hypoxia inducible factor (HIF) activation, dysregulation of angiogenesis, mitochondrial abnormalities and altered glucose and fatty acid transport/metabolism. It is important to understand how miRNAs are altered in these adverse environments to identify key pathways that can be targeted using miRNA mimics or inhibitors to condition an improved developmental response.

## 1. Introduction

Cardiovascular and metabolic disorders often present in adult life, but may have their origins in changes to the intrauterine environment during fetal development [1,2]. Given that the number of cardiomyocytes a human will have for life is set at birth, it is important to understand how cardiomyocyte endowment is regulated in the developing fetus. The importance of miRNA expression in cardiac development has been recognised, but how an adverse intrauterine environment can disrupt miRNA expression in the fetal heart and subsequently alter the critical process of heart development has not yet been explored. The prevalence of intrauterine growth restriction (IUGR) is as high as 10% worldwide [3] and the number of women of reproductive age who are classified as overweight/obese has increased to as many as 1 in 5 in pregnancy [4,5]. Therefore, it is important to determine the impact that these adverse fetal conditions have on cardiac health in adulthood. Herein we highlight a number of changes to the maternal and intrauterine environment and how these may impact on the expression of fetal cardiac miRNAs during development.

## 2. Developmental Programming in Early Life and Heart Disease

**Figure 1:**
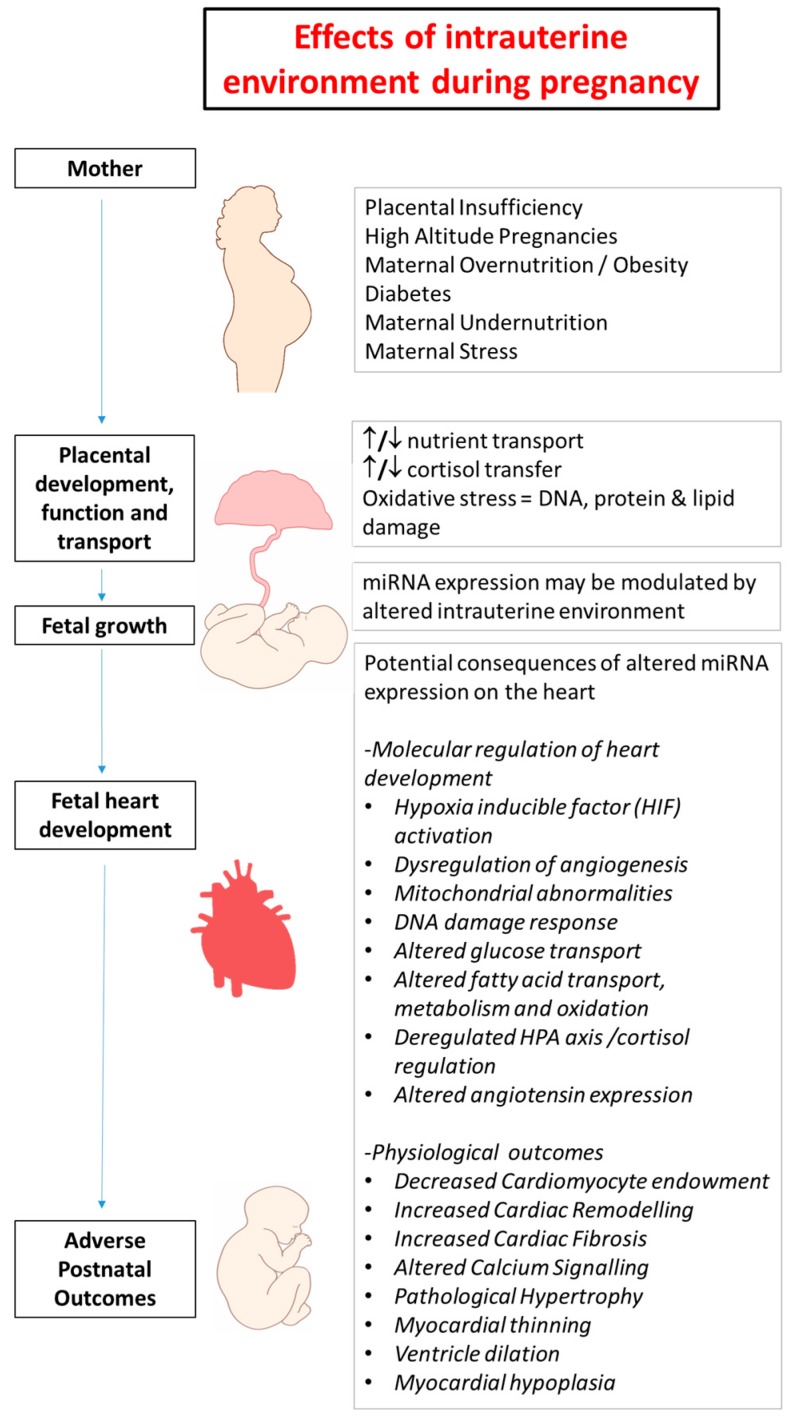
Summary of adverse intrauterine environments during pregnancy that may impact miRNA expression resulting in dysregulation of heart development and adverse postnatal physiological outcomes.

There were ~17.3 million deaths from cardiovascular disease in 2013, making it one of the leading causes of death globally [6,7,8]. Cardiovascular disease in adults is generally attributed to an unhealthy lifestyle with a focus on poor diet and smoking, interacting to a weak extent with genetic susceptibility [9,10]. However, emerging evidence suggests that cardiovascular and metabolic disorders in adult life can be initiated by physiological insults during fetal development (Figure 1) [1,2]. The associations between impaired development in utero and cardiovascular disease later in life can be explained by the concept of the developmental origins of health and disease (DOHaD) [11]. The DOHaD hypothesis is defined as the “setting” of a physiological system by an early stimulus or insult, during a critical period of development, when the manipulation of the environment, including oxygenation and nutritional factors, can have long-lasting, or programmed, consequences for the physiology of the fetus [1,11,12]. These insults can include placental insufficiency, living at high altitude, maternal obesity, diabetes, maternal undernutrition and stress, each of which can impact on the fetal intrauterine environment through changes in fetal oxygenation, nutrition and the hormonal environment. The DOHaD hypothesis may help explain how changes in utero can result in altered epigenetic regulation of cardiomyocyte endowment after an environmental insult and how this is subsequently linked to programming of cardiovascular disease in adult life.

## 3. Dysregulation of the Epigenome during Fetal Development 

**Figure 2:**
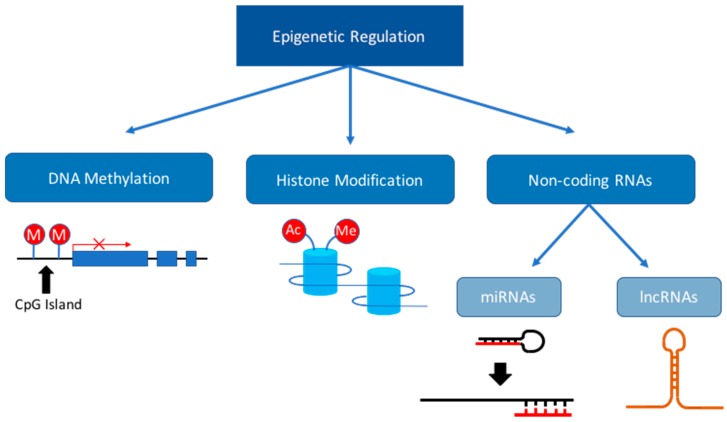
Epigenetic mechanisms. Epigenetic regulation can occur through several different pathways. These include DNA methylation, histone modification and non-coding RNAs. Non-coding RNAs such as miRNAs and lncRNAs are capable of modulating gene expression through suppression of mRNA expression or by direct regulation of epigenetic modifying enzymes.

Epigenetic mechanisms involving mitotically heritable changes in gene expression without changes to the DNA sequence can regulate fetal development and fetal programming during pregnancy [13]. The control of fetal programming is mediated by at least three epigenetic pathways; (i) DNA methylation, [14]; (ii) histone modifications that change how DNA is packaged with histone proteins to form chromatin and (iii) the expression of non-coding RNAs including miRNAs and long noncoding RNAs (lncRNAs; Figure 2) [13,14]. Specific miRNAs regulate the expression of genes encoding epigenetic modifier enzymes while some lncRNAs bind to specific DNA sites and provide scaffolds for binding of epigenetic modifier enzymes [15]. Epigenetic processes are tightly regulated during embryonic and fetal growth and have important roles in the normal development of organs, including the heart. Interactions between these epigenetic processes create the unique gene expression programs that orchestrate the development of each tissue and this is particularly important in cardiac development as many of these processes impact on metabolism. However, the epigenome is vulnerable to dysregulation throughout life by environmental factors; with development and embryogenesis being the most vulnerable periods [16]. Changes in the intrauterine environment can cause epigenetic dysregulation, which is termed “environmental epigenomics” [16]. This reflects the continuous interaction between the epigenome and the environment, including both endogenous (e.g., hormone levels or immune response) and exogenous factors (e.g., oxygenation, nutritional and drug exposures) [17]. The interplay between early life environmental epigenomics and development may lead to altered tissue function later in life. It is therefore likely that changes in the intrauterine environment, such as hypoxemia and dysregulation of maternal nutrient intake will impact upon the epigenome during pregnancy potentially generating lifelong consequences. This review will focus on fetal cardiac miRNA expression in the context of several adverse maternal conditions including; maternal or fetal hypoxia, obesity, undernutrition and stress, and the potential negative postnatal physiological outcomes. 

## 4. miRNA Expression is Essential for the Regulation of Cellular Function in the Heart

**Figure 3:**
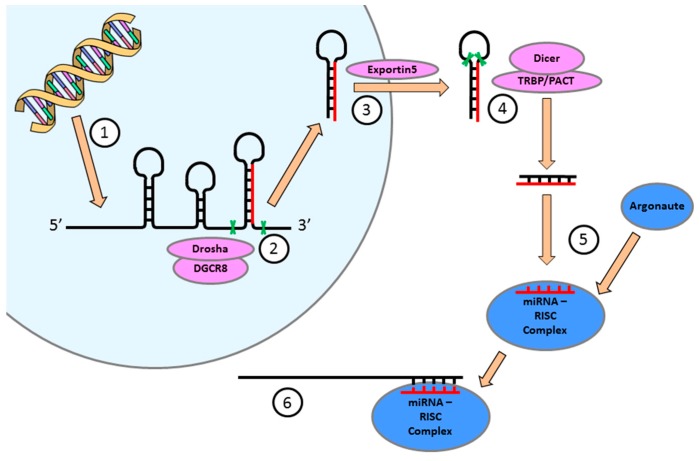
Processing of miRNAs: (1) miRNAs are transcribed as 5′-capped large polyadenylated precursor transcripts (pri-miRNA) primarily in a Polymerase II-dependent manner. Approximately 40% of human miRNAs are co-transcribed as clusters encoding up to eight distinct miRNA sequences in a single pri-miRNA transcript; (2) Pri-miRNAs are cleaved within the nucleus by the microprocessor complex containing Drosha (an RNaseIII-type nuclease) and a protein co-factor, DiGeorge syndrome critical region 8 gene (DGCR8) in humans; (3) The resulting 60–70 nucleotide hairpin structure (pre-miRNA) encodes for a single miRNA sequence that is exported from the nucleus to the cytoplasm by Exportin-5; (4) Cytoplasmic pre-miRNAs are further cleaved by another RNaseIII-nuclease, Dicer, in concert with cofactors (TRBP and PACT in humans), to eliminate the pre-miRNA loop sequence forming a short-lived asymmetric duplex intermediate (miRNA: miRNA*); (5) The mature miRNA is then loaded into the miRISC complex with Argonaute proteins; (6) miRNA guided suppression of the target mRNA. Identification of the target mRNA is assisted by the “seed sequence”, which is essential for the binding of the miRNA to the mRNA. The seed sequence is a conserved heptametrical sequence that is typically situated at positions 2–7 from the miRNA 5′-end. Even if the base pairing of miRNA with its target mRNA does not match perfectly, the “seed sequence” has to be perfectly complementary.

Epigenetics is involved in the control of fetal heart development via multiple pathways such as covalent modifications of DNA and histone proteins, as well as miRNA expression, which acts to repress gene expression by interfering with mRNA translation or stability. DNA methylation and histone modification not only regulate the expression of protein-encoding genes, but also miRNAs [18]. Conversely, a subset of miRNAs control the expression of other epigenetic regulators including DNA methyltransferases, histone deacetylases and polycomb group genes [18]. This feedback network between miRNAs and other epigenetic pathways forms an “epigenetics–miRNA regulatory circuit” that organizes the whole gene expression profile [18]. The human heart encodes over 700 miRNAs [19], which modulate the expression of more than 30% of the protein encoding genes [20]. These miRNAs are widely conserved across eukaryotes, and play an essential role in almost all aspects of cardiac development and postnatal heart function [21]. miRNA precursors are expressed in the nucleus (Figure 3) and this expression is often controlled in a cell type specific manner via transcription factors [22]. The miRNA profile of each organ, including the heart, is therefore unique. miRNA precursor transcripts are processed in the nucleus and cytoplasm (Figure 3) before the functional ~22 nucleotide miRNAs are formed. The mature miRNA then binds to the Argonaute protein to direct the RNA-induced silencing complex (Figure 3; RISC; also referred to as miRNA-ribonucleoprotein complex) to the target mRNA [22]. The miRISC complex regulates gene expression by translational repression or RISC-mediated cleavage of the mRNA. Many miRNAs are highly conserved between species and often retain the same mRNA targets. Since the human genome expresses a large variety of different miRNAs in heart tissue, and bioinformatics algorithms predict that each miRNA has hundreds of target transcripts, intricate regulation of essential cardiac developmental pathways is therefore possible [23].

Although the exact role of miRNAs in the epigenetic regulation of cardiac development has not been fully elucidated, the importance of these non-coding RNAs has been evaluated by manipulating the enzymes required to form the mature miRNA. Dicer, the enzyme required to process pre-miRNAs into functional miRNA, is encoded for by a single gene *Dicer1*. Mutations in the *Dicer1* gene are embryonically lethal at a gestational age of 7.5 days in rodents [24] and lead to generalized growth arrest in zebrafish embryos, which rarely survive beyond 13–14 days post-fertilization [25]. Due to the lethality of global *Dicer1* silencing, tissue specific deletion methods have helped determine the role of this enzyme in cardiac development. Conditional knockout models utilising Cre-recombinase in cardiac progenitor cells exhibit lethality or a poorly developed myocardium, indicating that cardiac specific *Dicer1* expression is essential in the morphogenesis and development of the fetal heart [26,27]. Dicer activity is also required for the maintenance of postnatal cardiac function, as demonstrated by cardiomyocyte-specific *Dicer1* ablation, which results in reduced mature miRNA in neonatal mouse heart and associated dilative cardiomyopathy and heart failure by postnatal day 4 [28]. Furthermore, Cre mediated cardiac deletion of *Dicer1* leads to spontaneous cardiac remodelling in three week old mice, resulting in increased rates of sudden death or a significant reduction in cardiac function by the end of week 4 [29]. *Dicer1* deletion in adult mice also results in cardiac remodeling. However, unlike their younger counterparts, adult mice do not display premature sudden death. Instead *Dicer1* deletion in adulthood results in severe histopathological changes including pathological hypertrophy, extensive inflammation and interstitial fibrosis [29]. *Dicer1* deletion therefore demonstrates the integral role of miRNAs in the regulation of cardiac development as well as postnatal maintenance of heart function.

Some of the most highly expressed miRNAs in the developing heart; miR-1 and miR-133a, have served as excellent examples of the regulatory requirement for miRNAs during heart development. In vivo studies have demonstrated the requirement for each of these miRNAs, with targeted deletion and knockdown resulting in either embryonic lethality, pericardial edema, ventricular septal defects or chamber dilatation in mouse models [30]. In addition to animal studies, several congenital cardiac defects have been associated with altered expression of specific miRNAs. Within the hearts of patients of the most common genetic disorder that leads to cardiac abnormalities, trisomy 21 (Down Syndrome), five miRNAs were found to be overexpressed (miR-99a, let-7c, miR-125b-2, miR-155 and miR-802), all of which are present on chromosome 21 [31]. Children with non-syndromic tetralogy of fallot have 61 miRNAs significantly changed in their myocardium compared to normally developing subjects [32]. Lastly, in the congenital heart condition of single ventricle malformation; 38 miRNAs were downregulated and 10 upregulated compared to normal control cardiac tissue, with WNT and mTOR signaling pathways as the most significantly affected by these changes [33]. These animal model and clinical studies provide a strong basis for the fundamental requirement of miRNA expression during fetal development, as well as demonstrate the consequences to their dysregulation in utero.

## 5. Chronic Hypoxemia Alters Heart Development and Changes the Expression of Cardiac miRNAs

Although fetal hearts have a remarkable ability to grow, function and survive in a low oxygen environment, chronic hypoxemia is associated with many cardiac related complications, and can cause both short and long term effects [34]. Chronic hypoxemia during fetal development can significantly delay heart growth and this is of particular importance in high altitude pregnancies, with ~140 million people living at high altitude worldwide [35]. Pregnant women living at high altitude have lower PaO_2_ (50 mm Hg) and thus there is altered placental growth compared to a pregnancy at sea level (PaO_2_, 95 mm Hg) [36,37]. These pregnancies are also at an increased risk of developing IUGR (birth weight < 10th centile for gestational age) and/or delivering a low birth weight (LBW; birth weight < 2.5 kg) infant, which has been related to increased infant mortality, premature birth, and development of cardiovascular related diseases in adulthood [38,39]. Chronic hypoxemia in utero is not limited to high altitude pregnancies, with additional causes that can include: umbilical cord compression, placental insufficiency resulting from aberrant placental development, pre-eclampsia, smoking, pollution and hemoglobinopathy [40]. LBW associated with reduced fetal growth is at least twice as common in developing countries, and reflects poor maternal nutrition, although maternal smoking is an important risk factor [40]. These factors contribute to reduced oxygen delivery and in some cases nutrients, to fetal tissues, which induces chronic hypoxemia in utero, resulting in adaptations to fetal development due to environmental stress. 

The adverse conditions created by chronic hypoxemia in utero are particularly disruptive to the development of cardiac tissue, due to its high metabolic demand [34]. Insufficient oxygen in utero results in myocardial thinning and ventricle dilation in addition to epicardium detachment in fetal rat hearts [41]. Similarly, hypoxemia delayed fetal heart maturation, when compared to controls in both chickens and mice [41,42], with either myocardial hypoplasia or cardiomyocyte hypertrophy in other animal models including sheep, as well as in humans [34,41,43,44,45,46,47]. The increased size of cardiomyocytes postnatally is likely to be compensatory for the reduced total number of cardiomyocytes [3,48,49] and reinforced by the increased heart to body weight ratio when exposed to prenatal hypoxemia [43,44,47]; suggesting either reduced growth of other non-essential organs in favour of heart development or cardiac enlargement. This decrease in cardiomyocyte number in hypoxemic fetuses is likely influenced via increased programmed cell death and autophagy during critical windows of heart development [48]. Hypoxia mediated apoptosis is controlled by increased cell death signalling through elevated caspase 3 activity and Fas activation, and suppressed by cell survival pathways, as indicated by Bcl-2 and Hsp70 expression in fetal hearts [44]. The reduction of proliferation is a result of premature exit from the cell cycle, as seen by the higher percentage of terminally differentiated cardiomyocytes in the heart at birth [44,50,51,52,53]. Prolonged insufficient oxygen supply can therefore cause abnormal fetal heart structural development, due to reduced cardiomyocyte proliferation and increased apoptosis and autophagy. 

**Figure 4:**
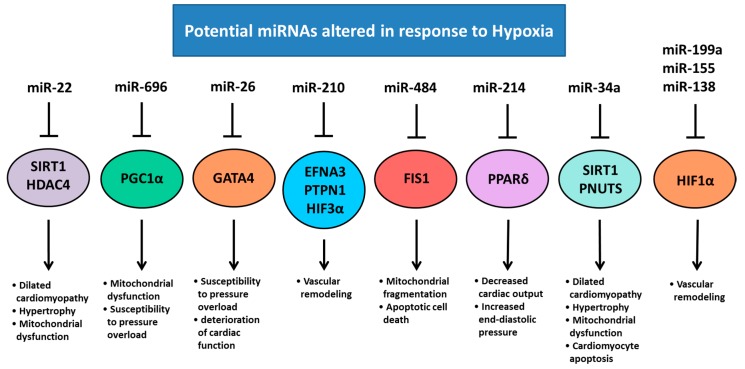
Regulation of miRNA expression in response to hypoxia. miR-138, miR-155 and miR-199a are induced in response to hypoxia, but can also modulate HIF1α; a major regulator of the hypoxia response and associated with vascular remodeling [54,76]. miR-22 regulates SIRT1 and HDAC4 expression that are associated with dilated cardiomyopathy, hypertrophy, and mitochondrial dysfunction [77]. miR-696 regulates PGC1α, dysfunction of this can result in mitochondrial dysfunction and increased susceptibility to pressure overload [78]. GATA4 plays an important role in the response pressure overload [79] and is a target of miR-26. miR-210 targets EFNA3, PTPN1 and HIF3α that have been associated with vascular remodeling [80]. miR-484 regulates expression of FIS1 which is associated with mitochondrial fragmentation and apoptotic cell death [81]. PPARδ plays a major role in the onset of cardiac failure [82] and is a target of miR-214. miR-34a also regulates SIRT1 as well as PNUTS that plays a role in DNA damage responses and cardiomyocyte apoptosis [61]. (SIRT1; Sirtuin 1, HDAC4; Histone deacetylase 4, PGC1α; PPARG coactivator 1 α, GATA4; GATA binding protein 4, EFNA3; Ephrin A3, PTPN1; Protein-tyrosine phosphatase 1B, HIF3α; Hypoxia inducible factor 3 α subunit, FIS1; Fission, Mitochondrial 1, PPARδ; Peroxisome proliferator activated receptor delta, PNUTS; Protein phosphatase 1 regulatory subunit 10). Pointed arrow represent downstream effects, blocked arrows represent downregulation of target genes.

The mechanisms underpinning the changes in gene expression and function of hypoxemic fetal hearts are complex and not completely understood, however, it is likely that miRNA expression plays a key role. The chief regulator of oxygen homeostasis in the hypoxemic heart are the hypoxia inducible factors (HIFs), which mediate the expression of genes with hypoxia response elements [48]. During acute hypoxia, HIFs recruit mechanisms to increase oxygen supply (angiogenesis, erythropoiesis and vasodilation) and decrease oxygen demand (decreased oxidative metabolism and increased glycolysis), as well as regulating the cell cycle, apoptosis and autophagy [48,54]. Although the mechanisms underpinning HIF stabilization in hypoxemia are well understood, many mechanisms involved in the regulation of its expression remain unclear. miRNAs have been implicated in the regulation of HIFs, with microarray analysis revealing several hypoxia-inducible miRNAs. Among these are miR-155, miR-138, miR-26, miR-22, miR-34a, miR-214, miR-199a, miR-696, miR-484, and miR-210 (Figure 4), which target HIFs, or have confirmed hypoxia response elements (HRE) in their promoter regions, implicating them in a network of miRNAs that may regulate or are regulated by HIFs [55,56,57,58,59,60,61,62,63,64,65,66,67]. Vascular endothelial growth factor (VEGF) also has a HRE and is a positive regulator of angiogenesis, as well as a pivotal growth factor in fetal development and is regulated by many factors [68], but the role of miRNAs in its regulation is not clear. Some miRNAs that have predicted targets to angiogenic factors are sharply down-regulated after hypoxia treatment in cell culture, including miR-15b, miR-16, miR-20a and miR-20b [69], suggesting that they are involved in VEGF regulation. Chronic hypoxemia can therefore alter the amount of myocyte proliferation, apoptosis and hypertrophy that occurs in the fetal heart [48], but the exact role of miRNA expression in this process is yet to be fully elucidated. 

Interestingly, a specific set of miRNAs are expressed almost exclusively in the placenta and secreted into the fetal circulation via exosomes [70,71,72]. These circulating miRNAs act in a similar mechanism to hormones, and can be detected in both the maternal and fetal compartments, including the fetal heart [72]. The expression of miRNAs was shown to be disrupted in placental tissue from small for gestational age pregnancies and placental pathologies such as IUGR [73,74] as well as mothers exposed to environmental pollutants [75]. The role of these placental specific miRNAs in heart development have yet to be fully explored, but there is some evidence that altered maternal nutrition as well as placental pathologies such as placental ablation, umbilical cord compression, and placental insufficiency alter circulating miRNA expression and this may impact fetal heart development.

## 6. Changes in Heart Development and miRNA Expression as a Result of Altered Maternal Nutrition or Diabetes

**Figure 5:**
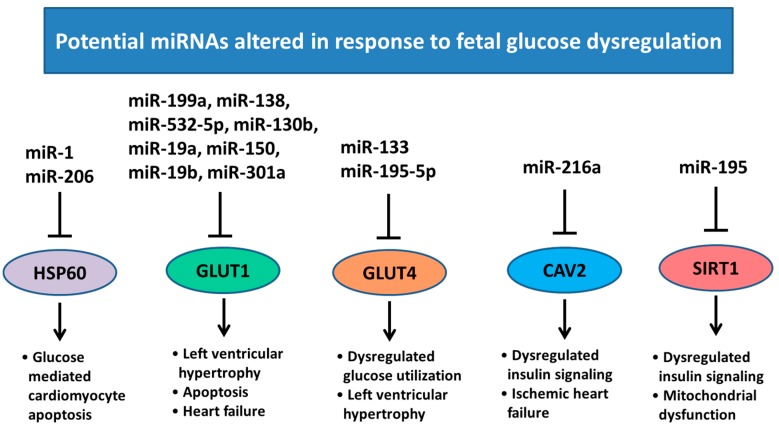
Regulation of miRNA expression in response to altered glucose transport. miR-199a, miR-138, miR-150, miR-532-5p, miR-130b, miR-19a, miR-19b and miR-301a have been implicated in regulating GLUT1 expression; associated with left ventricular hypertrophy and heart failure [96,97]. miR-133 and 195-5p regulate GLUT4 expression, which has been associated with hypertrophy and glucose homeostasis within the heart [98]. Expression of miR-1 and miR-206 are upregulated by increased glucose concentrations and target HSP60 what has a role in glucose mediated apoptosis in cardiomyocytes [99]. miR-216a regulates CAV2 expression, which plays a role in ischemic heart failure [94]. miR-195 expression is dysregulated by diabetes and acts on SIRT1, involved in insulin signaling and mitochondrial function [95]. HSP60; heat shock protein 60, GLUT1; glucose transporter 1, GLUT4; glucose transporter 4 CAV2; caveolin 2, SIRT1; sirtuin 1. Pointed arrow represent downstream effects, blocked arrows represent downregulation of target genes.

Low birth weight and undernutrition have been associated with increased risk of chronic heart disease in adult life [83,84,85]. This could possibly be linked to epigenetic adaptations that have occurred in utero to ensure survival of the fetus, but lead to adverse health outcomes in adulthood. Nutrition may be the largest non-genetic influence in fetal development, including factors such as maternal body composition, diet, and the efficiency of nutrient transport to the fetus [86]. The fetal heart is sensitive to changes in glucose availability as glucose oxidation is the main source of energy in the developing heart [87]. Throughout pregnancy there is a dominance of glucose transporter 1 (GLUT1) in cardiac tissue, however its expression can be modulated by maternal diet. In cell culture, *GLUT1* mRNA is down-regulated as a result of reduced nutrients, and this transcript has been identified as a target of several miRNAs such as miR-130b, miR-19a, miR-19b and miR-301a [88]. Conversely, when *GLUT1* is up-regulated as seen in cell culture models of maternal diabetes and maternal overnutrition, there is a correlation with decreased miR-199a, miR-138, miR-150 and miR-532-5p (Figure 5) [88]. After birth, glucose transport in the heart is mostly achieved by glucose transporter 4 (GLUT4) [89] and regulated by the insulin receptor (IR), insulin receptor substrate 1 (IRS-1) and PI3K, which in turn phosphorylates phosphoinositide-dependent kinase-1 (PDPK-1) and/or Akt. The activation of these processes can translocate GLUT4 to the plasma membrane to facilitate postnatal glucose uptake. *GLUT4* expression is regulated by miR-133 and miR-195-5p, both of which have been implicated in the inhibition of cardiomyocyte proliferation (Figure 5) [90]. High glucose concentrations also increase the expression of miR-1 and miR-206 in cardiomyocytes, which have been implicated in myocyte development [91] and high levels of apoptosis in H9c2 cells [92]. The diabetic heart experiences a number of metabolic changes characterized by insulin resistance, reduced cellular glucose import and oxidation, and increased mitochondrial fatty acid import and oxidation. A number of miRNA have been identified to play a role in regulating these metabolic changes including miR-216a, miR-199a, miR-195 and miR-34a [93,94]. In addition to metabolic changes, miRNAs may also play a role in diabetic cardiomyocyte apoptosis and cell survival signaling [95]. miRNA expression in the fetal heart may be significantly modulated by maternal nutrition and plasma glucose concentrations, and it is therefore important to identify the key miRNAs and their target genes, which are affected by this process and how this may influence cardiomyocyte endowment.

**Figure 6:**
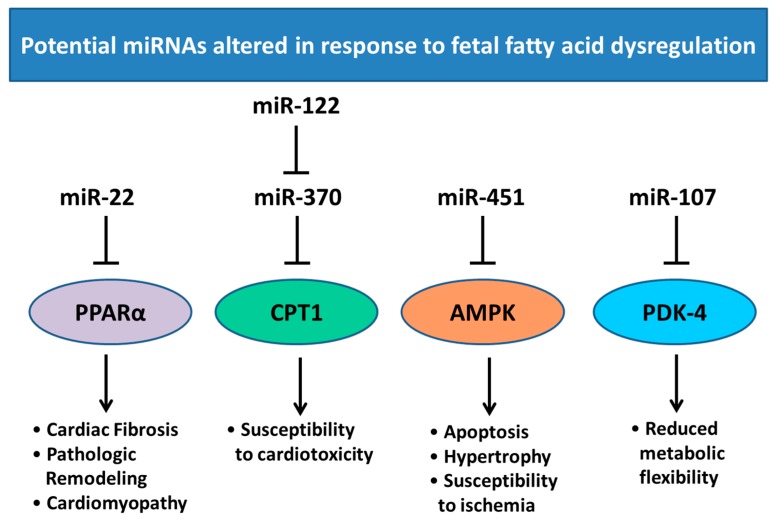
Regulation of miRNA expression in response to altered fatty acid dysregulation. PPARα has been associated with cardiac fibrosis, pathologic remodeling and cardiomyopathy during development [111] and is a target of miR-22 in the heart. miR-122 regulates miR-370 which in turn regulates CPT1 expression which has been associated with susceptibly to cardiotoxicity [112]. miR-451 mediates AMPK expression which is associated with apoptosis, hypertrophy and increased susceptibility to ischemia [113]. PDK-4 expression has been associated with loss of metabolic flexibility and exacerbation of cardiomyopathy [114] and is a predicted target of miR-107. PPARα; peroxisome proliferator activated receptor, CPT1; carnitine palmitoyltransferase-1, AMPK; AMP-activated protein kinase, PDK-4; pyruvate dehydrogenase kinase-4. Pointed arrow represent downstream effects, blocked arrows represent downregulation of target genes.

Shortly after birth there is a transition from mainly glucose metabolism to fatty acid oxidation as a source of cardiac energy, and the programmed effects acquired during fetal development may alter the expression of key postnatal factors in the regulation of fatty acid metabolism. Fatty acid oxidation in the heart is regulated by the phosphorylation of AMP-activated protein kinase (AMPK) and acetyl CoA carboxykinase (ACC) [100,101,102]. Whereas β-oxidation in the heart is also regulated by peroxisome proliferator activated receptor (PPARα; promotes uptake, utilization, and catabolism of fatty acids [103]), carnitine palmitoyltransferase-1 (CPT1; facilitates the transport of fatty acids into the mitochondria [104]), and pyruvate dehydrogenase kinase-4 (PDK-4; inhibits PDH, upregulating fatty acid β-oxidation [105,106]). *Cpt1α* expression is controlled by miR-370, which in turn is regulated by miR-122 (Figure 6) [107]. There are several other miRNAs associated with this pathway, including miR-451, which has higher expression in glucose rich environments, controlling *AMPK* expression [108]; miR-22, which regulates PPARα [109]; and miR-107, which regulates PDK-4 expression (Figure 6) [110]. Altered maternal nutrition may prime the fetal heart for exposure to stress in the postnatal environment, and it is therefore important to understand the underlying mechanisms that are regulated via miRNA expression. 

## 7. Altered Cardiac miRNA Expression in Response to Maternal Disease and Stress

**Figure 7:**
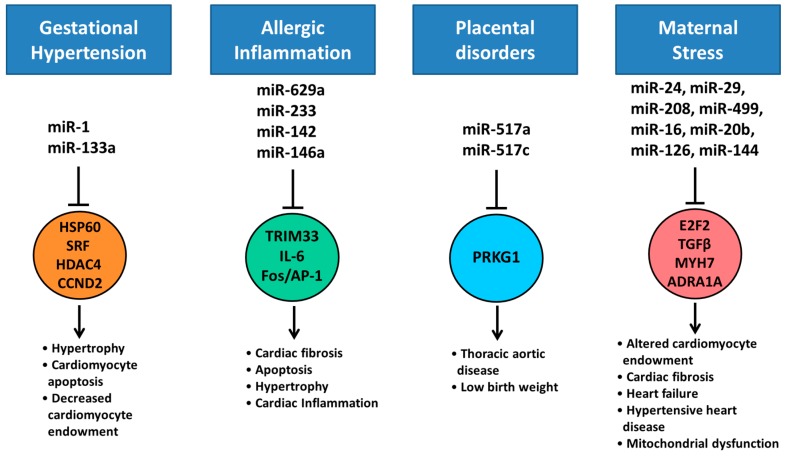
Regulation of miRNA expression in response to maternal disease and stress. HSP60, SRF, HDAC4 and CCND2 have been associated with cardiomyocyte hypertrophy, apoptosis and regulation of cardiomyocyte endowment [133] and are targets of miR-1 and miR-133a in the heart, that are dysregulated by maternal hypertension [91,115]. miR-629a regulates TRIM33 expression that in turn regulates the TGFβ/Smad signaling pathway which has been associated with cardiac fibrosis, hypertrophy and apoptosis [134]. miR-142 mediates a number of target genes including IL-6 [135], a potent myokine. The Fos/AP-1 pathway is a key component of cardiac inflammation and is a predicted target of miR-146a [136]. Placenta produced miR-517a has been associated with low birth fetuses and targets PRKG1 which has been associated with thoracic aortic disease [137,138]. miR-24 is a modulator of cell cycle, targeting E2F2 [139]. miR-29 targets TGFβ expression and has been associated with increased cardiac fibrosis after myocardial infarction [140]. miR-208 and miR-499 has been shown to regulate MYH7 a contractile subunit in the heart, and is associated with heart failure [141,142]. miR-16 targets ADRA1A and is associated with hypertensive heart disease [143]. GATA4 expression has been shown regulate miR-144 expression and modulate the response to ischemia/reperfusion cardiomyocyte injury [144]. miR-20b is an important regulator of apoptosis, differentiation and mitochondrial function [145]. HSP60; heat shock protein 60, SRF; serum response factor; HDAC4; histone deacetylase 4, CCND2; cyclin D2, TRIM33; tripartite motif containing 33, IL-6; interleukin 6, AP-1; activator protein 1, PRKG1; cGMP-dependent protein kinase 1, E2F2; E2F transcription factor 2, TGFβ; transforming growth factor β, MYH7; myosin heavy chain 7, ADRA1A; α-1A adrenergic receptor.

In addition to gestational diabetes (as discussed in Section 6), there are a number of diseases prevalent in pregnant women what may alter fetal miRNA expression. Gestational hypertension effects up to 8% of pregnancies and has been associated with a number of changes in miRNA expression in peripheral blood, including highly expressed cardiac miRNAs; miR-1 and miR-133a, (Figure 7) and have a strong correlation with severity of disease [115]. These circulating miRNAs can transfer across the placenta to regulate fetal gene expression [116]. Maternal infections during pregnancy often pose a significant threat to the heath of the fetus. Individual viral, bacterial and parasitic infections have specific sets of miRNAs associated with their physiological response [117,118]. Although the current dogma is that the fetus is kept sterile from maternal bacterial infection, miRNAs that are altered in response to maternal infection can transfer to the fetus, altering fetal gene expression. In addition, viruses can also gain access to the placenta and fetus through a variety of means, including transmission through trophoblasts, transfer of infected macrophages, invasive surgical procedures or vaginal infection [119]. Maternal inflammation from allergy may also play a role in transfer of circulating miRNAs to the fetus. Asthma for example has been associated with a specific subset of miRNAs in biological fluids, including miR-629-3p, miR-223-3p, miR-142-3p, and miR-146a (Figure 7) [120,121]. 

Placental disorders remain as one of the largest impacts on fetal development, including those found in ectopic pregnancies, placenta previa, intrauterine bleeding and placental abruption. Previous studies have proven distinct dysregulation of placental miRNAs in response to these disorders. For example, placenta previa and placental abruption have both been associated with altered circulating concentrations of miR-517a and miR-517c (Figure 7) [122], pregnancy-associated, placenta-specific miRNAs. As discussed above, these placentally expressed miRNAs are released into the fetal circulation via exosomes and act somewhat like hormones, though the effect on cardiac development has not yet been explored [70,71,72]. It is also important to note that these disorders may also interrupt placental transfer of maternal miRNAs to the fetal tissues, impairing any exosomal miRNA signaling that may be important to fetal development.

Maternal stress can be caused by a range of factors including psychological stress (e.g., grief, anxiety and depression) and physical stress (e.g., shift work, undernutrition or environmental mishaps) [123,124]. If sufficiently severe, these factors may alter the intrauterine environment, pre-emptively priming the fetus for an anticipated stressful postnatal environment. Maternal stress during pregnancy has been extensively studied in relation to the development of the fetal brain and the hypothalamo–pituitary–adrenal (HPA) axis. These studies demonstrate changes in molecular and behavioral differences in offspring of chronically stressed mothers [123,125,126,127]. The major stress hormone, cortisol, increases in the maternal circulation two to four fold over the course of gestation. Fetal exposure to the increasing concentrations of maternal cortisol is regulated by 11BHSD2 in the placenta, which acts to convert active cortisol into the inactive form cortisone [127]. The expression of 11BHSD2 in the placenta also increases across gestation, providing partial protection of the fetus from maternal cortisol. However, the placental expression of this enzyme decreases towards the end of pregnancy, allowing a large portion of maternal cortisol to reach the fetus. This normal decrease in placental 11BHSD2 expression works in concert with the developing fetal adrenal gland to ensure that the fetus has sufficient cortisol during the third trimester of pregnancy, when many organs—especially the lungs—require this for complete maturation. Although the fetus is somewhat protected from maternal cortisol for most of the time during gestation, in some situations where cortisol is over-secreted or placental transport is impaired, excess levels of maternal cortisol can reach the fetus. 

Several miRNAs have been identified as regulators of steroid production and the stress response. For example, miR-24 is a key regulator of *CYP11B1* (11β-hydroxylase) and *CYP11B2* (aldosterone synthase), responsible for the final stages of cortisol and aldosterone biosynthesis [128]. Several other miRNAs, including miR-29, miR-208, miR-499, miR-16, miR-20b, miR-126 and miR-144, (Figure 7) have altered cardiac expression or circulating concentrations in response to stress [129,130,131]. High concentrations of cortisol during pregnancy can negatively impact on heart development with evidence of hypertrophy in myocytes from the left ventricular free wall from fetal sheep and altered glucose transport and angiotensin expression [132]. It is likely that miRNAs play a role in these important changes. However, the expression of miRNAs in heart tissue under high cortisol stimulation during fetal development is yet to be investigated in detail. 

## 8. Conclusions

Cardiovascular and metabolic disorders in adult life may derive their origins from changes to the intrauterine environment during fetal development. Given that the number of cardiomyocytes a human will have for life is set at birth, it is important to understand how cardiomyocyte endowment is regulated during early fetal life, which may lead to novel therapies. Intrauterine growth restriction and fetal hypoxemia can negatively impact heart development and the effects may persist into adult life. Dysregulation of miRNA pathways as a result of hypoxia may contribute to these negative outcomes. Although currently the molecular pathways resulting in adverse outcomes have not been elucidated in overweight, obese or diabetic pregnancies, we have identified key pathways by which changes to glucose metabolism may dysregulate the expression of key miRNAs involved in developing a healthy heart. Since there are thousands of miRNAs, each regulating multiple target genes, this complex network of gene regulation will require further investigation to tease apart the impact of each altered miRNA on their regulatory networks. Taken together, it is clear that changes in miRNA expression as a result of altered intrauterine environment may contribute to negative cardiac outcomes as a result of fetal hypoxemia, maternal overnutrition and maternal stress. Future research may identify key miRNAs that can be targeted using mimics or inhibitors to condition an improved response in adverse developmental conditions.
